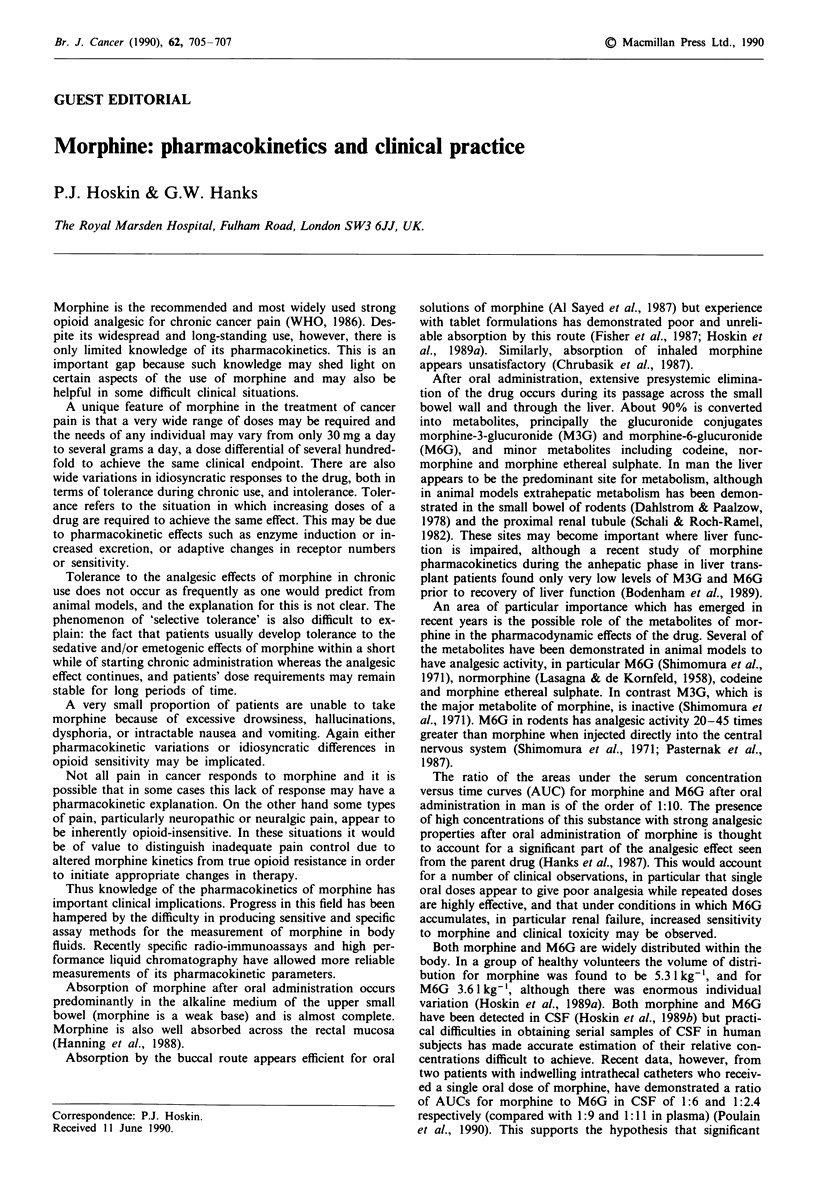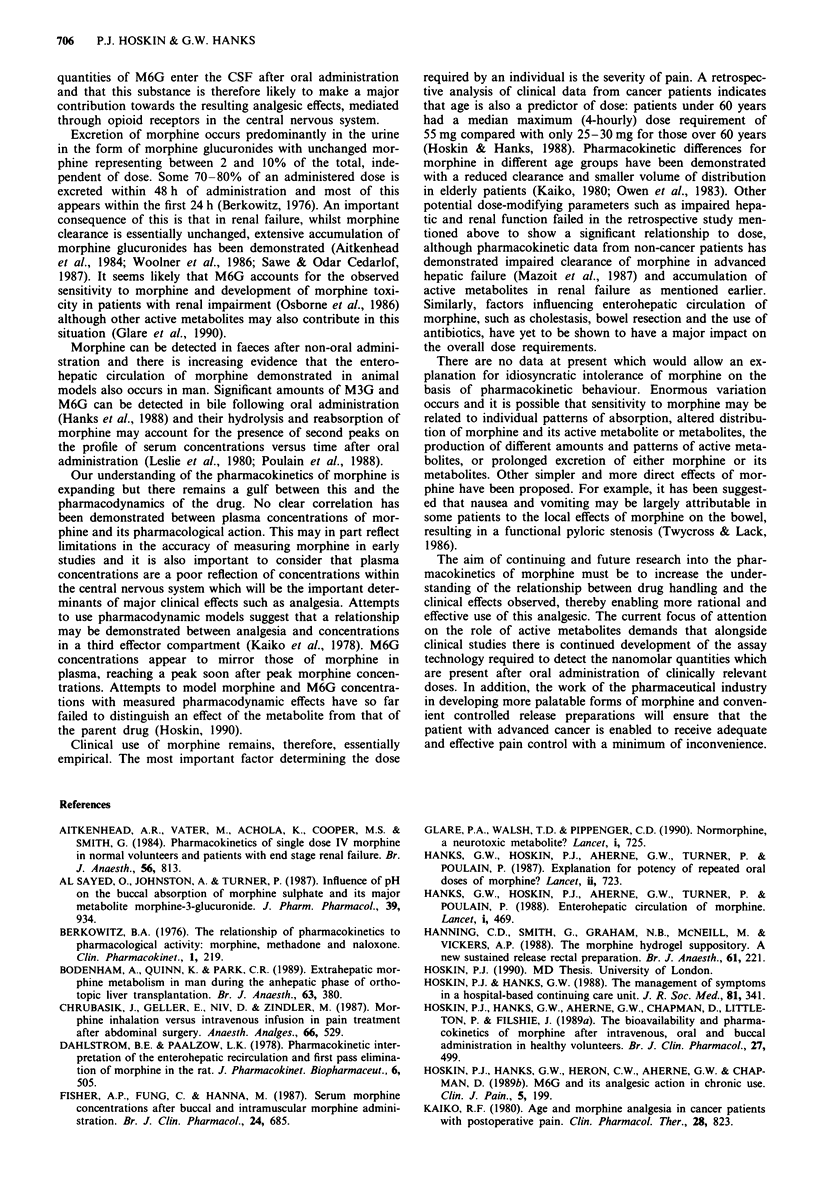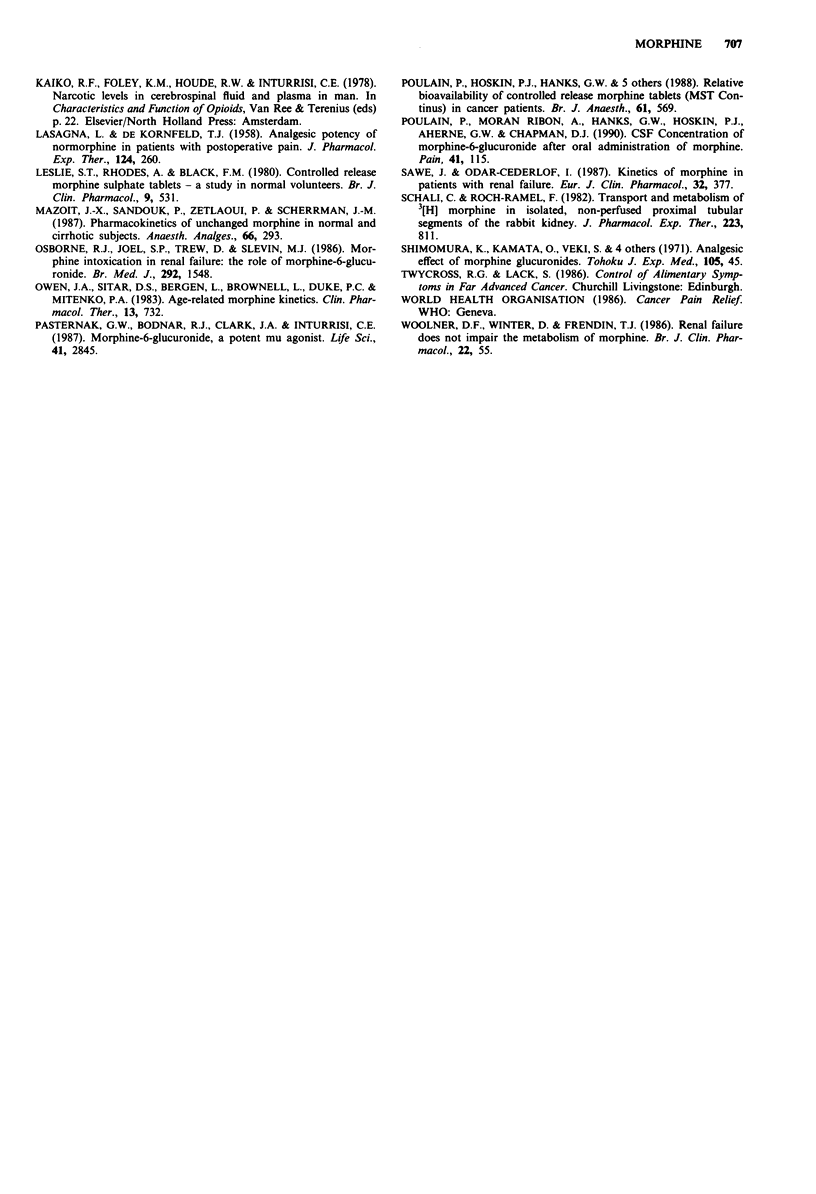# Morphine: pharmacokinetics and clinical practice.

**DOI:** 10.1038/bjc.1990.363

**Published:** 1990-11

**Authors:** P. J. Hoskin, G. W. Hanks


© Macmillan Press Ltd., 1990

GUEST EDITORIAL

### Morphine: pharmacokinetics and clinical practice

P.J. Hoskin & G.W. Hanks

*The Royal Marsden Hospital, Fulham Road, London SW3 6JJ, UK.*

Morphine is the recommended and most widely used strong opioid analgesic for chronic cancer pain (WHO, 1986). Despite its widespread and long-standing use, however, there is only limited knowledge of its pharmacokinetics. This is an important gap because such knowledge may shed light on certain aspects of the use of morphine and may also be helpful in some difficult clinical situations.

A unique feature of morphine in the treatment of cancer pain is that a very wide range of doses may be required and the needs of any individual may vary from only 30 mg a day to several grams a day, a dose differential of several hundredfold to achieve the same clinical endpoint. There are also wide variations in idiosyncratic responses to the drug, both in terms of tolerance during chronic use, and intolerance. Tolerance refers to the situation in which increasing doses of a drug are required to achieve the same effect. This may be due to pharmacokinetic effects such as enzyme induction or increased excretion, or adaptive changes in receptor numbers or sensitivity.

Tolerance to the analgesic effects of morphine in chronic use does not occur as frequently as one would predict from animal models, and the explanation for this is not clear. The phenomenon of 'selective tolerance' is also difficult to explain: the fact that patients usually develop tolerance to the sedative and/or emetogenic effects of morphine within a short while of starting chronic administration whereas the analgesic effect continues, and patients' dose requirements may remain stable for long periods of time.

A very small proportion of patients are unable to take morphine because of excessive drowsiness, hallucinations, dysphoria, or intractable nausea and vomiting. Again either pharmacokinetic variations or idiosyncratic differences in opioid sensitivity may be implicated.

Not all pain in cancer responds to morphine and it is possible that in some cases this lack of response may have a pharmacokinetic explanation. On the other hand some types of pain, particularly neuropathic or neuralgic pain, appear to be inherently opioid-insensitive. In these situations it would be of value to distinguish inadequate pain control due to altered morphine kinetics from true opioid resistance in order to initiate appropriate changes in therapy.

Thus knowledge of the pharmacokinetics of morphine has important clinical implications. Progress in this field has been hampered by the difficulty in producing sensitive and specific assay methods for the measurement of morphine in body fluids. Recently specific radio-immunoassays and high performance liquid chromatography have allowed more reliable measurements of its pharmacokinetic parameters.

Absorption of morphine after oral administration occurs predominantly in the alkaline medium of the upper small bowel (morphine is a weak base) and is almost complete. Morphine is also well absorbed across the rectal mucosa (Hanning *et al.*, 1988).

Absorption by the buccal route appears efficient for oral solutions of morphine (Al Sayed *et al.*, 1987) but experience with tablet formulations has demonstrated poor and unreliable absorption by this route (Fisher *et al.*, 1987; Hoskin *et al.*, 1989*a*). Similarly, absorption of inhaled morphine appears unsatisfactory (Chrubasik *et al.*, 1987).

After oral administration, extensive presystemic elimination of the drug occurs during its passage across the small bowel wall and through the liver. About 90% is converted into metabolites, principally the glucuronide conjugates morphine-3-glucuronide (M3G) and morphine-6-glucuronide (M6G), and minor metabolites including codeine, normorphine and morphine ethereal sulphate. In man the liver appears to be the predominant site for metabolism, although in animal models extrahepatic metabolism has been demonstrated in the small bowel of rodents (Dahlstrom & Paalzow, 1978) and the proximal renal tubule (Schali & Roch-Ramel, 1982). These sites may become important where liver function is impaired, although a recent study of morphine pharmacokinetics during the anhepatic phase in liver transplant patients found only very low levels of M3G and M6G prior to recovery of liver function (Bodenham *et al.*, 1989).

An area of particular importance which has emerged in recent years is the possible role of the metabolites of morphine in the pharmacodynamic effects of the drug. Several of the metabolites have been demonstrated in animal models to have analgesic activity, in particular M6G (Shimomura *et al.*, 1971), normorphine (Lasagna & de Kornfeld, 1958), codeine and morphine ethereal sulphate. In contrast M3G, which is the major metabolite of morphine, is inactive (Shimomura *et al.*, 1971). M6G in rodents has analgesic activity 20-45 times greater than morphine when injected directly into the central nervous system (Shimomura *et al.*, 1971; Pasternak *et al.*, 1987).

The ratio of the areas under the serum concentration versus time curves (AUC) for morphine and M6G after oral administration in man is of the order of 1:10. The presence of high concentrations of this substance with strong analgesic properties after oral administration of morphine is thought to account for a significant part of the analgesic effect seen from the parent drug (Hanks *et al.*, 1987). This would account for a number of clinical observations, in particular that single oral doses appear to give poor analgesia while repeated doses are highly effective, and that under conditions in which M6G accumulates, in particular renal failure, increased sensitivity to morphine and clinical toxicity may be observed.

Both morphine and M6G are widely distributed within the body. In a group of healthy volunteers the volume of distribution for morphine was found to be 5.3 l kg$^{-1}$, and for M6G 3.6 l kg$^{-1}$, although there was enormous individual variation (Hoskin *et al.*, 1989*a*). Both morphine and M6G have been detected in CSF (Hoskin *et al.*, 1989*b*) but practical difficulties in obtaining serial samples of CSF in human subjects has made accurate estimation of their relative concentrations difficult to achieve. Recent data, however, from two patients with indwelling intrathecal catheters who received a single oral dose of morphine, have demonstrated a ratio of AUCs for morphine to M6G in CSF of 1:6 and 1:2.4 respectively (compared with 1:9 and 1:11 in plasma) (Poulain *et al.*, 1990). This supports the hypothesis that significant

Correspondence: P.J. Hoskin.
Received 11 June 1990.

quantities of M6G enter the CSF after oral administration and that this substance is therefore likely to make a major contribution towards the resulting analgesic effects, mediated through opioid receptors in the central nervous system.

Excretion of morphine occurs predominantly in the urine in the form of morphine glucuronides with unchanged morphine representing between 2 and 10% of the total, independent of dose. Some 70–80% of an administered dose is excreted within 48 h of administration and most of this appears within the first 24 h (Berkowitz, 1976). An important consequence of this is that in renal failure, whilst morphine clearance is essentially unchanged, extensive accumulation of morphine glucuronides has been demonstrated (Aitkenhead *et al.*, 1984; Woolner *et al.*, 1986; Sawe & Odar Cedarlof, 1987). It seems likely that M6G accounts for the observed sensitivity to morphine and development of morphine toxicity in patients with renal impairment (Osborne *et al.*, 1986) although other active metabolites may also contribute in this situation (Glare *et al.*, 1990).

Morphine can be detected in faeces after non-oral administration and there is increasing evidence that the enterohepatic circulation of morphine demonstrated in animal models also occurs in man. Significant amounts of M3G and M6G can be detected in bile following oral administration (Hanks *et al.*, 1988) and their hydrolysis and reabsorption of morphine may account for the presence of second peaks on the profile of serum concentrations versus time after oral administration (Leslie *et al.*, 1980; Poulain *et al.*, 1988).

Our understanding of the pharmacokinetics of morphine is expanding but there remains a gulf between this and the pharmacodynamics of the drug. No clear correlation has been demonstrated between plasma concentrations of morphine and its pharmacological action. This may in part reflect limitations in the accuracy of measuring morphine in early studies and it is also important to consider that plasma concentrations are a poor reflection of concentrations within the central nervous system which will be the important determinants of major clinical effects such as analgesia. Attempts to use pharmacodynamic models suggest that a relationship may be demonstrated between analgesia and concentrations in a third effector compartment (Kaiko *et al.*, 1978). M6G concentrations appear to mirror those of morphine in plasma, reaching a peak soon after peak morphine concentrations. Attempts to model morphine and M6G concentrations with measured pharmacodynamic effects have so far failed to distinguish an effect of the metabolite from that of the parent drug (Hoskin, 1990).

Clinical use of morphine remains, therefore, essentially empirical. The most important factor determining the dose required by an individual is the severity of pain. A retrospective analysis of clinical data from cancer patients indicates that age is also a predictor of dose: patients under 60 years had a median maximum (4-hourly) dose requirement of 55 mg compared with only 25–30 mg for those over 60 years (Hoskin & Hanks, 1988). Pharmacokinetic differences for morphine in different age groups have been demonstrated with a reduced clearance and smaller volume of distribution in elderly patients (Kaiko, 1980; Owen *et al.*, 1983). Other potential dose-modifying parameters such as impaired hepatic and renal function failed in the retrospective study mentioned above to show a significant relationship to dose, although pharmacokinetic data from non-cancer patients has demonstrated impaired clearance of morphine in advanced hepatic failure (Mazoit *et al.*, 1987) and accumulation of active metabolites in renal failure as mentioned earlier. Similarly, factors influencing enterohepatic circulation of morphine, such as cholestasis, bowel resection and the use of antibiotics, have yet to be shown to have a major impact on the overall dose requirements.

There are no data at present which would allow an explanation for idiosyncratic intolerance of morphine on the basis of pharmacokinetic behaviour. Enormous variation occurs and it is possible that sensitivity to morphine may be related to individual patterns of absorption, altered distribution of morphine and its active metabolite or metabolites, the production of different amounts and patterns of active metabolites, or prolonged excretion of either morphine or its metabolites. Other simpler and more direct effects of morphine have been proposed. For example, it has been suggested that nausea and vomiting may be largely attributable in some patients to the local effects of morphine on the bowel, resulting in a functional pyloric stenosis (Twycross & Lack, 1986).

The aim of continuing and future research into the pharmacokinetics of morphine must be to increase the understanding of the relationship between drug handling and the clinical effects observed, thereby enabling more rational and effective use of this analgesic. The current focus of attention on the role of active metabolites demands that alongside clinical studies there is continued development of the assay technology required to detect the nanomolar quantities which are present after oral administration of clinically relevant doses. In addition, the work of the pharmaceutical industry in developing more palatable forms of morphine and convenient controlled release preparations will ensure that the patient with advanced cancer is enabled to receive adequate and effective pain control with a minimum of inconvenience.

### References

AITKENHEAD, A.R., VATER, M., ACHOLA, K., COOPER, M.S. & SMITH, G. (1984). Pharmacokinetics of single dose IV morphine in normal volunteers and patients with end stage renal failure. *Br. J. Anaesth.*, **56**, 813.

AL SAYED, O., JOHNSTON, A. & TURNER, P. (1987). Influence of pH on the buccal absorption of morphine sulphate and its major metabolite morphine-3-glucuronide. *J. Pharm. Pharmacol.*, **39**, 934.

BERKOWITZ, B.A. (1976). The relationship of pharmacokinetics to pharmacological activity: morphine, methadone and naloxone. *Clin. Pharmacokinet.*, **1**, 219.

BODENHAM, A., QUINN, K. & PARK, C.R. (1989). Extrahepatic morphine metabolism in man during the anhepatic phase of orthotopic liver transplantation. *Br. J. Anaesth.*, **63**, 380.

CHRUBASIK, J., GELLER, E., NIV, D. & ZINDLER, M. (1987). Morphine inhalation versus intravenous infusion in pain treatment after abdominal surgery. *Anaesth. Analges.*, **66**, 529.

DAHLSTROM, B.E. & PAALZOW, L.K. (1978). Pharmacokinetic interpretation of the enterohepatic recirculation and first pass elimination of morphine in the rat. *J. Pharmacokinet. Biopharmaceut.*, **6**, 505.

FISHER, A.P., FUNG, C. & HANNA, M. (1987). Serum morphine concentrations after buccal and intramuscular morphine administration. *Br. J. Clin. Pharmacol.*, **24**, 685.

GLARE, P.A., WALSH, T.D. & PIPPENGER, C.D. (1990). Normorphine, a neurotoxic metabolite? *Lancet*, **i**, 725.

HANKS, G.W., HOSKIN, P.J., AHERNE, G.W., TURNER, P. & POULAIN, P. (1987). Explanation for potency of repeated oral doses of morphine? *Lancet*, **ii**, 723.

HANKS, G.W., HOSKIN, P.J., AHERNE, G.W., TURNER, P. & POULAIN, P. (1988). Enterohepatic circulation of morphine. *Lancet*, **i**, 469.

HANNING, C.D., SMITH, G., GRAHAM, N.B., McNEILL, M. & VICKERS, A.P. (1988). The morphine hydrogel suppository. A new sustained release rectal preparation. *Br. J. Anaesth.*, **61**, 221.

HOSKIN, P.J. (1990). MD Thesis. University of London.

HOSKIN, P.J. & HANKS, G.W. (1988). The management of symptoms in a hospital-based continuing care unit. *J. R. Soc. Med.*, **81**, 341.

HOSKIN, P.J., HANKS, G.W., AHERNE, G.W., CHAPMAN, D., LITTLETON, P. & FILSHIE, J. (1989*a*). The bioavailability and pharmacokinetics of morphine after intravenous, oral and buccal administration in healthy volunteers. *Br. J. Clin. Pharmacol.*, **27**, 499.

HOSKIN, P.J., HANKS, G.W., HERON, C.W., AHERNE, G.W. & CHAPMAN, D. (1989*b*). M6G and its analgesic action in chronic use. *Clin. J. Pain.*, **5**, 199.

KAIKO, R.F. (1980). Age and morphine analgesia in cancer patients with postoperative pain. *Clin. Pharmacol. Ther.*, **28**, 823.

KAIKO, R.F., FOLEY, K.M., HOUDE, R.W. & INTURRISI, C.E. (1978). Narcotic levels in cerebrospinal fluid and plasma in man. In *Characteristics and Function of Opioids*, Van Ree & Terenius (eds) p. 22. Elsevier/North Holland Press: Amsterdam.

LASAGNA, L. & DE KORNFELD, T.J. (1958). Analgesic potency of normorphine in patients with postoperative pain. *J. Pharmacol. Exp. Ther.*, **124**, 260.

LESLIE, S.T., RHODES, A. & BLACK, F.M. (1980). Controlled release morphine sulphate tablets – a study in normal volunteers. *Br. J. Clin. Pharmacol.*, **9**, 531.

MAZOIT, J.-X., SANDOUK, P., ZETLAOUI, P. & SCHERRMAN, J.-M. (1987). Pharmacokinetics of unchanged morphine in normal and cirrhotic subjects. *Anaesth. Analges.*, **66**, 293.

OSBORNE, R.J., JOEL, S.P., TREW, D. & SLEVIN, M.J. (1986). Morphine intoxication in renal failure: the role of morphine-6-glucuronide. *Br. Med. J.*, **292**, 1548.

OWEN, J.A., SITAR, D.S., BERGEN, L., BROWNELL, L., DUKE, P.C. & MITENKO, P.A. (1983). Age-related morphine kinetics. *Clin. Pharmacol. Ther.*, **13**, 732.

PASTERNAK, G.W., BODNAR, R.J., CLARK, J.A. & INTURRISI, C.E. (1987). Morphine-6-glucuronide, a potent mu agonist. *Life Sci.*, **41**, 2845.

POULAIN, P., HOSKIN, P.J., HANKS, G.W. & 5 others (1988). Relative bioavailability of controlled release morphine tablets (MST Continus) in cancer patients. *Br. J. Anaesth.*, **61**, 569.

POULAIN, P., MORAN RIBON, A., HANKS, G.W., HOSKIN, P.J., AHERNE, G.W. & CHAPMAN, D.J. (1990). CSF Concentration of morphine-6-glucuronide after oral administration of morphine. *Pain*, **41**, 115.

SAWE, J. & ODAR-CEDERLOF, I. (1987). Kinetics of morphine in patients with renal failure. *Eur. J. Clin. Pharmacol.*, **32**, 377.

SCHALI, C. & ROCH-RAMEL, F. (1982). Transport and metabolism of $^3$[H] morphine in isolated, non-perfused proximal tubular segments of the rabbit kidney. *J. Pharmacol. Exp. Ther.*, **223**, 811.

SHIMOMURA, K., KAMATA, O., VEKI, S. & 4 others (1971). Analgesic effect of morphine glucuronides. *Tohoku J. Exp. Med.*, **105**, 45.

TWYCROSS, R.G. & LACK, S. (1986). *Control of Alimentary Symptoms in Far Advanced Cancer*. Churchill Livingstone: Edinburgh.

WORLD HEALTH ORGANISATION (1986). *Cancer Pain Relief*. WHO: Geneva.

WOOLNER, D.F., WINTER, D. & FRENDIN, T.J. (1986). Renal failure does not impair the metabolism of morphine. *Br. J. Clin. Pharmacol.*, **22**, 55.